# REST, regulated by RA through miR-29a and the proteasome pathway, plays a crucial role in RPC proliferation and differentiation

**DOI:** 10.1038/s41419-018-0473-5

**Published:** 2018-04-18

**Authors:** Yuyao Wang, Dandan Zhang, Zhimin Tang, Yi Zhang, Huiqin Gao, Ni Ni, Bingqiao Shen, Hao Sun, Ping Gu

**Affiliations:** 1 Department of Ophthalmology, Ninth People’s Hospital, Shanghai Jiao Tong University School of Medicine, 200011 Shanghai, P.R. China; 2 Shanghai Key Laboratory of Orbital Diseases and Ocular Oncology, 200011 Shanghai, P.R. China

## Abstract

One of the primary obstacles in the application of retinal progenitor cells (RPCs) to the treatment of retinal degenerative diseases, such as age-related macular degeneration (AMD) and retinitis pigmentosa (RP), is their limited ability to proliferate and differentiate into specific retinal neurons. In this study, we revealed that repressor element-1-silencing transcription factor (REST), whose expression could be transcriptionally and post-transcriptionally mediated by retinoic acid (RA, one isomeride of a vitamin A derivative used as a differentiation-inducing agent in many disease treatments), plays a pivotal role in the regulation of proliferation and differentiation of RPCs. Our results show that direct knockdown of endogenous REST reduced RPC proliferation but accelerated RPC differentiation toward retinal neurons, which phenocopied the observed effects of RA on RPCs. Further studies disclosed that the expression level of REST could be downregulated by RA not only through upregulating microRNA (miR)-29a, which directly interacted with the 3′-untranslated region (3′-UTR) of the REST mRNA, but also through promoting REST proteasomal degradation. These results show us a novel functional protein, REST, which regulates RPC proliferation and differentiation, can be mediated by RA. Understanding the mechanisms of REST and RA in RPC fate determination enlightens a promising future for the application of REST and RA in the treatment of retinal degeneration diseases.

## Introduction

Retinal progenitor cells (RPCs) are a side branch of neural progenitor cells that sustain the undifferentiated status with the potential for self-renewal and differentiation into retinal neuronal cells and have great potential to treat retinal degenerative diseases, such as age-related macular degeneration (AMD) and retinitis pigmentosa (RP)^1–3^. Although RPCs are identified as one source of replaceable multipotent progenitor cells that can be derived from both embryonic and adult mammalian retina, the limited proliferation and differentiation capacity toward specific retinal neurons in vitro restricts their future clinical applications^4–6^. This emphasizes the importance of a better understanding of the mechanisms controlling RPC proliferation and differentiation.

Repressor element-1 (RE-1) is known as a piece of conserved DNA sequence locating in the transcriptional regulatory regions of many neuronal genes^7–9^. Repressor element-1-silencing transcription factor (REST, also known as neuron-restrictive silencer factor, NRSF) is a zinc-finger protein, which interacts with RE-1, exerting a gene silencing effect^10–13^. Generally, during embryonic stem cells (ESCs) differentiation into neural progenitor cells (NPCs), REST is downregulated by proteasomal degradation. When transitioning from progenitor to mature neurons, REST and its corepressors dissociate from neuronal gene chromatin accompanied by its transcriptional repression^11,12^. The gradually decreased expression of REST is necessary during neuron differentiation and REST overexpression leads to neuronal gene expression disorder and axon pathfinding mistakes^14^. The literature suggests that REST plays an important role in the generation of functional mature neurons. Recently, REST has become a hot topic in the field of the central nervous system (CNS), as its expression levels in neurons closely correlate with recognition, longevity and neuropathological processes^15^. REST expression accumulates in seizures and epilepsy^16^, ischemia^17,18^ and alcoholism^19^, and relative phenotypes of these diseases can be attenuated by blocking REST function. On the other hand, in aging and Alzheimer’s disease, REST exerts a neuronal protective effect through spurring stress response genes expression and suppressing genes that facilitate cell death and disease pathology^15^. Although REST has been researched extensively in the CNS, its role in neural retina remains to be explored. As RPCs are one of the promising cell resources in the treatment of retinal degeneration diseases, it is worth detecting the role of REST in RPC fate determination.

Retinoic acid (RA) is a vitamin A derivative that is synthesized by the enzyme retinaldehyde dehydrogenase^20^ and plays a major role during the early development of the nervous system^21^. The mechanisms of RA that influence cell fates during the development of the nervous system have been investigated in many studies, including in the retina^22–27^. All-trans RA (ATRA) is an isomeride of RA and has been used to treat many kinds of diseases, that is, acne or other disorders of keratinization^28^, and, most importantly, to treat cancer, as it generally inhibits tumor cells proliferation and induces their differentiation and apoptosis^29^. The most established use of ATRA is in the induction of remission in patients with acute promyelocytic leukemia (APL)^30^. Moreover, the use of ATRA-based chemotherapy in the induction phase of the treatment of APL was approved by the Food and Drug Administration (FDA), which made it possible to use ATRA as a potential medicine to treat retinal degeneration.

The microRNA (miRNA) is one of small non-coding RNAs, which downregulate gene expression mainly through binding to their 3′-untranslated region (3′-UTR), causing translation repression or mRNA degradation^31^. miR-29a is one member of miR-29 family, which are involved in normal tissue differentiation^32^, including skeletal myogenesis^33^, osteoblast differentiation^34^ and neuron differentiation^35,36^. In addition, our previous study demonstrated that miR-29a plays an important role during RPC proliferation and differentiation by targeting RBM8a^37^.

In this study, the role of REST in the proliferation and differentiation of RPCs was investigated. We further identified the endogenous REST expression levels that could be mediated by exogenous RA through promoting miR-29a expression (to inhibit REST mRNA levels) and accelerating REST proteasomal degradation.

These results provide us with a deeper understanding of the mechanisms regarding how REST regulate RPCs’ self-renewal and differentiation and the promising field of using RA in the clinical treatment of retinal degeneration diseases.

## Materials and methods

### Cell isolation and culture

RPCs were obtained from the fresh neural retina that isolated from postnatal day 1 C57BL/6 mice^38^, and cells from the fifth passage or sixth passage were used in this study. The proliferation medium contains 20 ng/ml recombinant epidermal growth factor (EGF, Invitrogen, Carlsbad, CA, USA), advanced Dulbecco’s modified Eagle’s medium (DMEM)/F12 (Invitrogen), 2 mM l-glutamine (Invitrogen) and 1% N2 neural supplement (Invitrogen)^6^. The differentiation medium consists of 10% fetal bovine serum (FBS, Invitrogen), advanced DMEM/F12 (Invitrogen), 1% N2 neural supplement and without EGF.

All of the animals were handled according to the animal usage standards of the Association for Research in Vision and Ophthalmology (ARVO) and following approval by the Animal Research Committee of the Ninth People’s Hospital, Shanghai Jiao Tong University School of Medicine.

### Drug treatment

In our pre-experiment, cells were incubated with RA (Sigma-Aldrich, St. Louis, MO, USA) with a concentration from 10 to 500 ng/ml, and 100 ng/ml was chosen as the desired concentration (data not shown). RA was dissolved in dimethyl sulfoxide (DMSO, Sigma-Aldrich) with recommended storage at a concentration of 10 mM and stored at −20 °C in the dark. Individual aliquots were diluted to the appropriate concentration in the culture medium.

RPCs were cultured with 100 ng/ml RA in proliferation or differentiation medium according to the different experimental objectives. To detect the effects of RA on REST expression, RPCs were treated with RA (100 ng/ml) under proliferation medium for 3 days followed by the addition of 20 µM MG-132 (Sigma-Aldrich) for another 8 h. RPCs treated with or without RA for 3 days were used as the control.

### Luciferase assay

For the construction of the luciferase reporter vector, the 3′-UTR-wt (Genechem, Shanghai, China) of the REST mRNA was composed of a 713-bp fragment of the REST mRNA 3′-UTR containing the predicted miR-29a binding site (positions 561–567), and the REST mRNA 3′-UTR-mu (Genechem) was composed of REST mRNA 3′-UTR containing the predicted miR-29a mutant sequence.

HEK 293 cells were transfected with the miR-29a mimics or control miRNA. After 48 h, cells were used for luciferase assays according to the manufacturer’s instruction.

### Transfection

Small interfering RNAs, including siREST and negative control, miR-29a oligonucleotides, including the miR-29a inhibitor, miR-29a mimics and the negative control, were synthesized by Biomics Biotech Co., Ltd (Nantong, China). Before transient transfections, RPCs were cultured under differentiation medium to allow for attachment for 8–12 h. Then, Lipofectamine 2000 (Invitrogen) and oligonucleotides (siREST, miR-29a inhibitor, and negative control) were blended with Opti-MEM (Invitrogen) medium for 5 min, respectively. Following incubation of Lipofectamine 2000-Opti-MEM with oligonucleotide-Opti-MEM for 20 min^38^. RPCs were incubated with this mixture in plates for 6–8 h before it was replaced by proliferation or differentiation medium. Oligonucleotides were repeatedly transfected into RPCs every 3 days under differentiation culture conditions. The oligonucleotide sequence of siREST was as follows: 5′- GCGCUAAGAAGUUCUUUGUdTdT-3′.

### Quantification of the cells’ viability

The different groups of RPCs were cultured in 96-well plates with the density of 1×10^4^ per well. RPCs were incubated with Lipofectamine 2000 (control group) or siREST (siREST group); DMSO (control group) or 100 ng/ml RA (RA group); and Lipofectamine 2000 plus DMSO (control group), 100 ng/ml RA (RA group), 100 ng/ml RA plus miR-29a inhibitor (RA + miR-29a inhibitor group) or miR-29a (miR-29a group) under proliferation medium, and the cell counting kit (CCK-8, Dojindo, Kumamoto, Japan) was used to quantitate the number of viable cells. The CCK-8 solution was added to each well at different time points: 0 h, 24 h, 48 h and 72 h. The absorbance at 450 nm was measured using a microplate reader (ELX800, BioTek, Vermont, USA) after incubation for another 4 h at 37 °C.

### Reverse transcription and quantitative polymerase chain reaction (qPCR)

In all, 1 µg of total RNA was extracted and reverse transcribed according to previous study^38^. Then, the qPCR was performed with a 7500 Real-Time PCR Detection System (Applied Biosystems, Irvine, CA, USA) using 2 µl of 10-fold diluted complementary DNA, 10 µl of 2*miRcute miRNA premix (Tiangen Biotech Co.) or 10 µl of 2 × Power SYBR Green PCR Master Mix (Applied Biosystems), 2 µl (300 nM) primers of target genes (Table 1) and the rest was supplemented with nuclease-free water (Invitrogen) to make the reaction volume to be 20 µl. The relative mRNA was analyzed using the Pfaffl method after 40 cycles of amplification. The relative mRNA was expressed as the fold change relative to sonRNA-202 or β-actin, which was used as an endogenous normalization control for the miRNA and mRNA, respectively.

**Table 1:** Primers used for qPCR

Gene	Accession no.	Forward (5′-3′)	Reverse (5′-3′)	Annealing temperature [°C]	Product size (base pairs)
Ki-67	X82786	cagtactcggaatgcagcaa	cagtcttcaggggctctgtc	60	170
Nestin	NM_016701	aactggcacctcaagatgt	tcaagggtattaggcaagggg	60	235
Pax-6	NM_013627	agtgaatgggcggagttatg	acttggacgggaactgacac	60	269
β3-Tublin	NM_023279	cgagacctactgcatcgaca	cattgagctgaccagggaat	60	152
Rhodopsin	NM_145383	tcaccaccaccctctacaca	tgatccaggtgaagaccaca	60	216
Recoverin	NM_009038	atggggaatagcaagagcgg	gagtccgggaaaaacttggaata	60	184
Brn3a	NM_0111434	cgctctcgcacaacaacatga	ttcttctcgccgccgttga	60	121
Calbindin	NM_009788	ggcttcatttcgacgctgac	acgtgagccaactctacaattc	60	184
REST	NM_005612	gtgcgaactcacacaggaga	aagaggtttaggcccgttgt	60	201
Casepase-3	NM_004346	catggaagcgaatcaatggact	ctgtaccagaccgagatgtca	60	139
β-Actin	NM_007393	agccatgtacgtagccatcc	ctctcagctgtggtggtgaa	60	152

### Western blot analysis

Western blot analyses were performed as previously described^38^. Various antibodies, including rabbit monoclonal anti-REST (Abcam, Cambridge, UK), mouse anti-nestin (BD, San Jose, CA, USA), rabbit monoclonal anti-Pax-6 (Biolegend, San Diego, CA, USA), mouse monoclonal anti-β3-tubulin (Millipore, Billerica, MA, USA), mouse monoclonal anti-rhodopsin, rabbit polyclonal anti-recoverin (Millipore), mouse monoclonal anti-Brn3a (Millipore) and mouse monoclonal anti-Caspase-3 (Santa Cruz, California, USA) were diluted 1:1000, and mouse anti-β-actin (Sigma-Aldrich) was diluted 1:5000. The membranes were incubated with 1:5000 dilutions of DyLightTM680-conjugated goat anti-mouse or goat anti-rabbit secondary antibodies (Sigma-Aldrich), and analyzed by Odyssey V 3.0 image scanning (LI-COR, Lincoln, NE, USA).

### Immunocytochemistry

Digested RPCs were seeded on glass coverslips (VWR, West Chester, PA, USA) in 24-well plates. Cells treated with siREST or RA were cultured for 3 or 7 days in proliferation or differentiation medium, respectively. Then, cells were fixed with 4% paraformaldehyde (Sigma-Aldrich) for 20 min and treated with blocking reagent consisting of 10 % of goat serum (Sigma-Aldrich), 0.3% of Triton-100 (Sangon, Shanghai, China) in phosphate-buffered saline buffer. After 1 h, cells were incubated with various antibodies, including rabbit polyclonal anti-REST (Abcam), mouse monoclonal anti-Ki-67 (BD), mouse monoclonal anti-Nestin (Millipore), mouse monoclonal anti-β3-tubulin (Millipore) and rabbit polyclonal anti-recoverin (Millipore) overnight at 4 °C and incubated with Alexa Fluor546-goat anti-mouse/rabbit secondary antibodies (BD) in the dark for 1 h. Cell nuclei were counterstained with Hoechst (Invitrogen). Negative controls were performed in parallel but without primary antibodies. For 5-Bromo-2-deoxyuridine (BrdU) incorporation, the anti-BrdU antibody (Cell Signaling Technology, Danvers, MA, USA) was used to detect proliferating cells, which was treated with 10 mM BrdU (Sigma-Aldrich) for 10 h. Immunoreactive cells were visualized by the fluorescence microscope (Olympus BX51, Japan) and images of six random fields of each sample per experiment were taken to calculate the mean value. The mean values of three independent experiments of each group were used for statistical analysis.

### Live/dead assay

Live/dead assay was performed to detect cell viability using the LIVE/DEAD™ Viability/Cytotoxicity Kit (Life Technologies GmbH, Darmstadt, Germany) according to the product description^39,40^. Images of six random fields of each sample per experiment were taken by the fluorescence microscope (Olympus) to calculate the mean value. The mean values of three independent experiments of each group were used for statistical analysis.

### Statistical analyses

All experiments in our studies were repeated three times except for otherwise specified. Statistics were display with the mean ± standard derivation. The data were statistically analyzed by the one-way analysis of variance (ANOVA) or Student’s* t*-test, and the significant difference was set at *P* ≤ 0.05.

## Results

### Knockdown of endogenous REST inhibits RPC proliferation but promotes differentiation

**Fig. 1:**
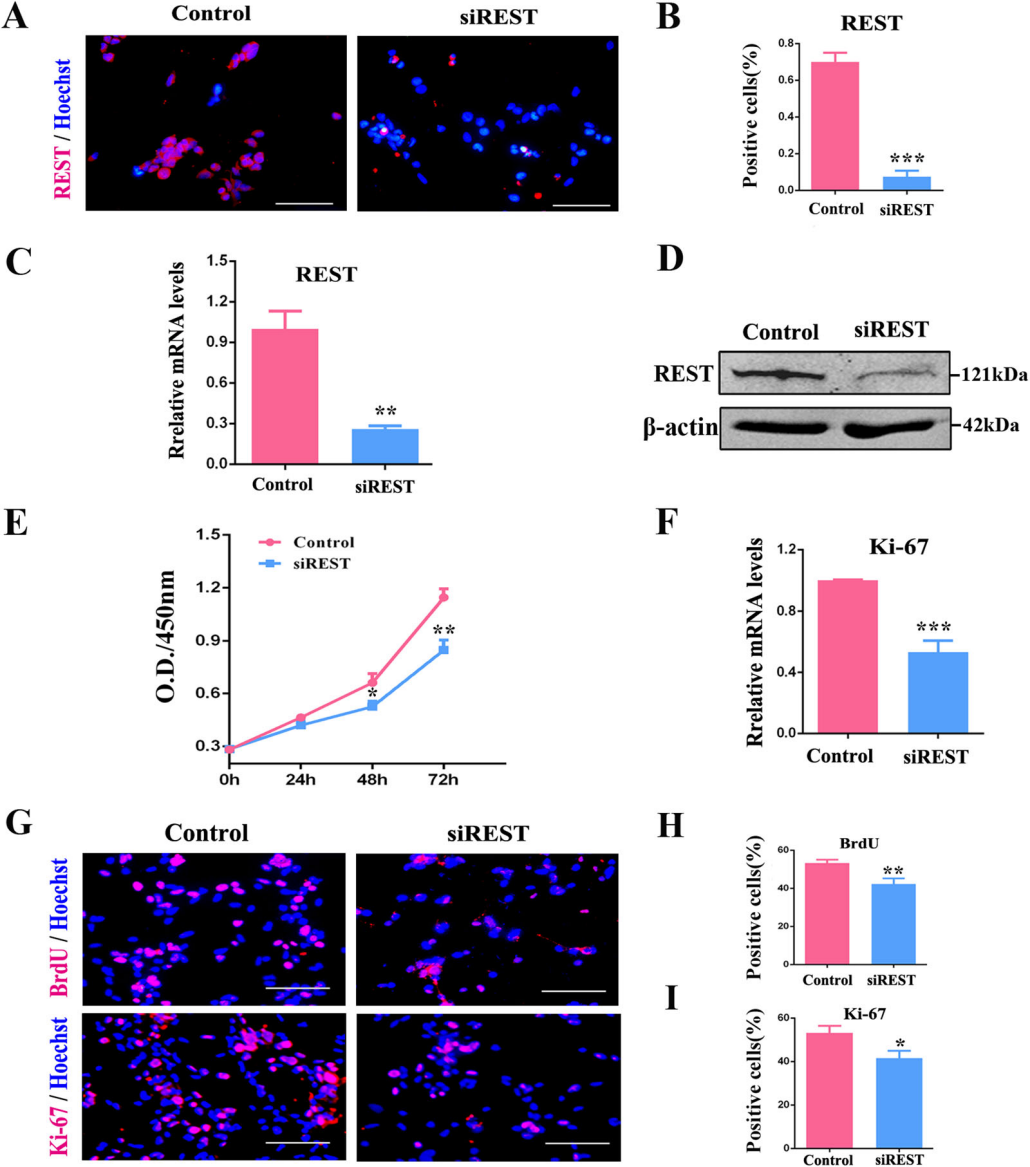
siREST inhibits RPC proliferation. **a**, **b** The immunocytochemistry, **c** qPCR analysis and **d** western blot results revealed that the expression levels of REST decreased in the siREST-treated RPC cultures compared with the control. **e** The proliferation ability of the RPCs was assessed via CCK-8 analysis. The proliferation ability of the RPCs markedly decreased after treatment with siREST under proliferation conditions. **f** According to the qPCR analysis, expression levels of Ki-67 decreased significantly in siREST-treated RPC cultures. **g**-**i** Immunocytochemistry with antibodies against BrdU and Ki-67 revealed the effects of siREST on RPC proliferation and was consistent with the results shown above. Scale bars: 100 μm. Data are the averages of three independent experiments. Error bars indicate the standard error of the mean. *P ≤ 0.01, **P ≤ 0.01, ***P ≤ 0.001 (Student’s *t-*test)

REST represses differentiation of ESCs, NPCs, bone marrow stromal cells, etc.^11,41^. However, the role of REST in the regulation of RPC fate is still unclear. To investigate the roles of REST in RPC proliferation and differentiation, we first detected the expression profile of REST in RPCs. As shown in Fig. 1a, REST protein was generally present in the nuclei of undifferentiated RPCs (under proliferation medium), and a significant reduction of REST expression was recorded after the siRNA of REST was designed and transfected into RPCs (Figs. 1a-d). Following Cell Counting Kit-8 (CCK-8) analysis, there was significantly inhibited expansion in siREST-treated cultures for the following 48 h and 72 h, while there was no marked difference between the two groups on the first day (Fig. 1e), implying knockdown of the expression of REST reduce RPC proliferation. Further, the qPCR results showed that the expression levels of Ki-67 (a marker for cell proliferation ability) decreased notably (approximately 50%) in siREST-treated RPC cultures compared with the control group (Fig. 1f). Then, the immunocytochemistry results showed that over 50% of the RPCs in the control group were positive for the cell proliferation markers BrdU and Ki-67 (Figs. 1g-i), which is indicative of active proliferation. The percentages of cells positive for BrdU and Ki-67 in the siREST group were measured to be approximately 42.25 ± 1.35 % and 41.57 ± 2.77%, respectively, which were significantly lower than those in the control group (Figs. 1g-i). However, compared with the control group, the percentage of nestin-positive cells exhibited no significant change although the immunoreactive intensity of nestin faded in siREST-treated RPC cultures (Fig. S1A-C). These results suggest that REST is important for sustaining RPC proliferation and interference of REST expression did not result in a loss of RPC multipotency.

**Fig. 2:**
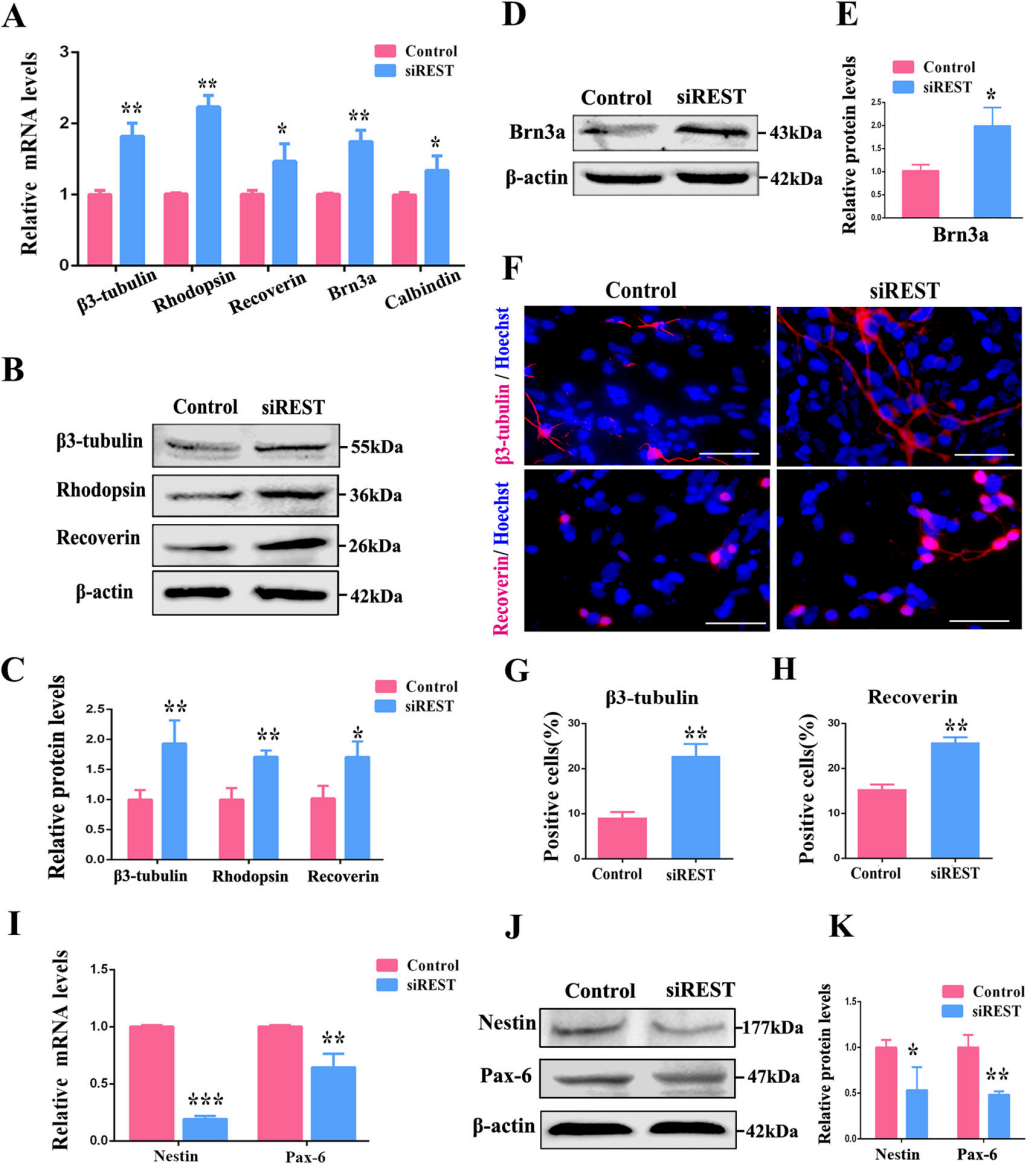
siREST enhances RPC differentiation. **a** The expression levels of RPC differentiation-related markers, including β3-tubulin (a pan-neuronal marker), rhodopsin (a marker for rod photoreceptors), recoverin (a marker for rod and cone photoreceptors), Brn3a (a marker for ganglion neurons) and calbindin (a marker for horizontal neurons), were elevated by siREST according to qPCR analysis. **b**–**e** The results of western blot showed that the expression levels of β3-tubulin, rhodopsin, recoverin and Brn3a were augmented in siREST-treated RPC cultures compared with the control. The western blot was scanned and normalized to β-actin. **f**-**h** Immunocytochemistry showed that the percentages of β3-tubulin- and recoverin-positive cells were markedly increased in the siREST-treated RPC cultures compared with the control. **i** qPCR results indicated that nestin and Pax-6 (retinal progenitor markers) were downregulated in siREST-treated RPC cultures. **j**-**k** The expression levels of nestin and Pax-6 were repressed after siREST treatment, according to the western blot. The western blot was scanned and normalized to β-actin. Scale bars: 50 μm. Data are the averages of three independent experiments. Error bars indicate the standard error of the mean. *P ≤ 0.05, **P ≤ 0.01, ***P ≤ 0.001 (Student’s *t-*test)

Further study was focused on the effects of REST on RPC differentiation. Before detection of RPC differentiation markers, such as β3-tubulin (a pan-neuronal marker), rhodopsin (the rod photoreceptor marker), recoverin (the cone and rod photoreceptor marker), Brn3a (a marker of ganglion neurons) and calbindin (a marker for horizontal neurons), RPCs were cultured in differentiation medium without (control group) or with siREST (siREST group) treatment for 7 days. In the RPC cultures treated with siREST, the expression levels of β3-tubulin, rhodopsin, recoverin, Brn3a and calbindin were obviously increased (Fig. 2a). The results of the western blot were consistent with our qPCR assay (Figs. 2b-e). In addition, immunocytochemistry analysis showed a marked increase in the β3-tubulin and recoverin marker levels upon siREST treatment (Figs. 2f-h), indicating that knockdown of REST expression enhanced RPC neuronal differentiation. In the meanwhile, we detected the progenitor markers nestin and Pax-6. The prominent decrease of nestin and Pax-6 expression implied that the process of differentiation was accelerated in siREST-treated cultures (Figs. 2i-k). Above all, these results suggest that REST plays a crucial role in RPC proliferation and differentiation.

### RA inhibits RPC proliferation and accelerates RPC differentiation

**Fig. 3:**
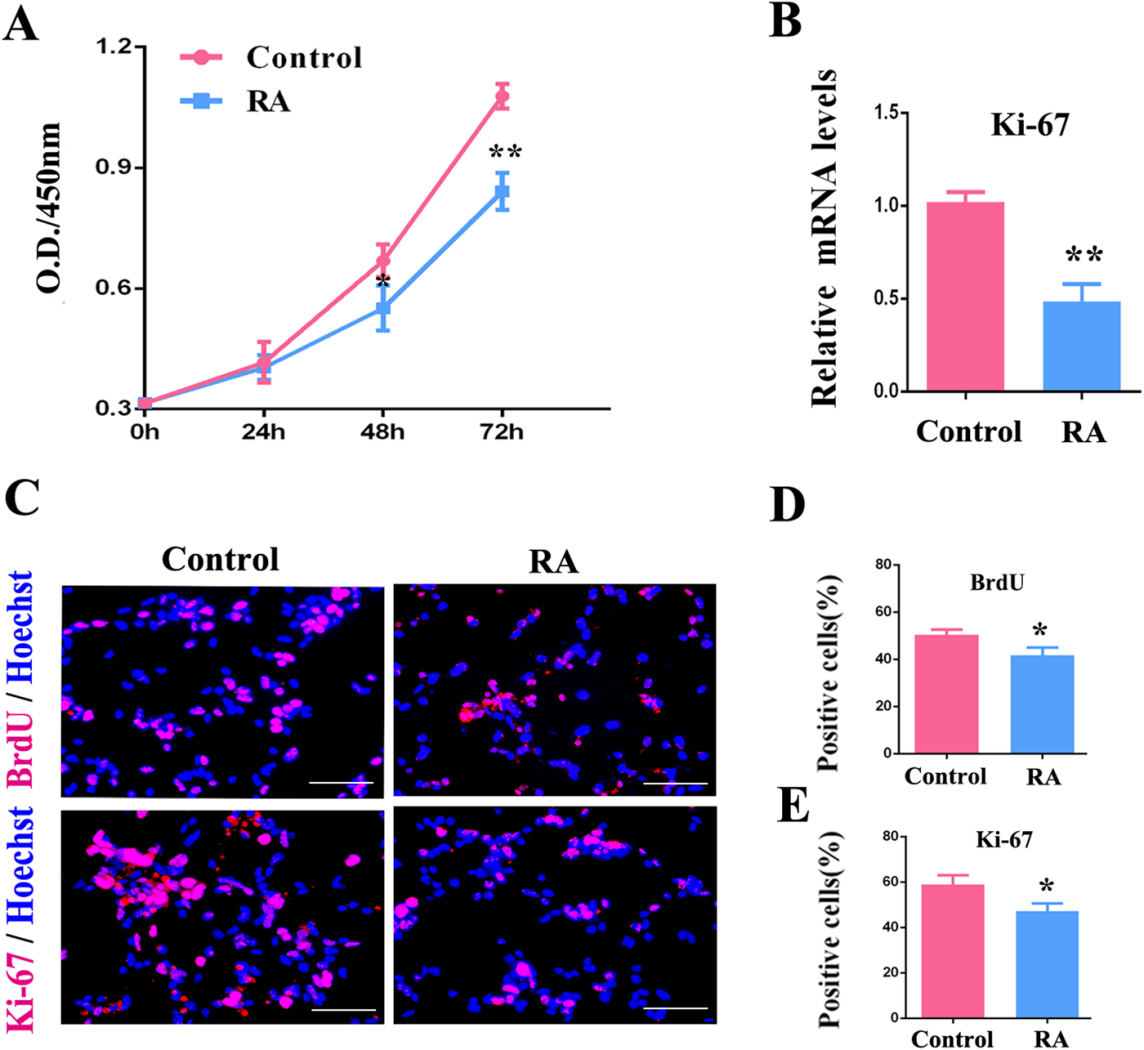
RA inhibits RPC proliferation. **a** The proliferation ability of the RPC cultures was assessed via CCK-8 analysis. A weakened proliferation capacity was detected in the RA-treated RPCs when compared with control. **b**The qPCR results revealed that the expression level of Ki-67 decreased in the RPC cultures treated with RA. **c**-**e** Immunochemistry showed that the percentages of BrdU- and Ki-67-positive cells in the RA-treated RPC cultures were significantly lower than those in the control group. Scale bars: 100 μm. Data are the averages of three independent experiments. Error bars indicate the standard deviation of the mean. *P ≤ 0.05, **P ≤ 0.01 (Student’s *t*-test)

REST is a repressor of neuronal genes whose expression is associated with poor neuronal differentiation^42^. As RA promotes neuronal differentiation, it may involve the modulation of REST expression. To detect the relationship between REST and RA in RPCs, the effects of RA on RPCs proliferation and differentiation were firstly determined. It is acknowledged that RA promoted photoreceptor differentiation in embryonic retinal and early postnatal retinal cultures^43^. In this study, we detected the effects of RA on RPC proliferation and differentiation. To evaluate the effects of RA on RPC proliferation, RPCs were cultured with 100 ng/ml RA for 3 days under proliferation medium. The suppression on RPC expansion was detected by CCK-8 and qPCR analysis, as shown in Figs. 3a, b. Additionally, as revealed in the immunocytochemistry analysis, the percentages of BrdU-positive and Ki-67-positive cells were significantly decreased in RA-treated RPC cultures (Figs. 3c-e). In addition, our data showed that the immunoreactive intensity of nestin in RA-treated RPC cultures is faded; however, the percentages of nestin-positive cells exhibited no significant difference between RA and control groups (Fig. S1D-F). Meanwhile, our data from qPCR and western blot showed that the significant difference of caspase-3 (a marker for cell apoptosis) expression level was not detected between RA and control group, which was consistent with our live/dead assay (Fig. S2). These results implied that RA inhibited the proliferation ability of RPCs without inducing cell apoptosis and reducing their multipotency.

**Fig. 4:**
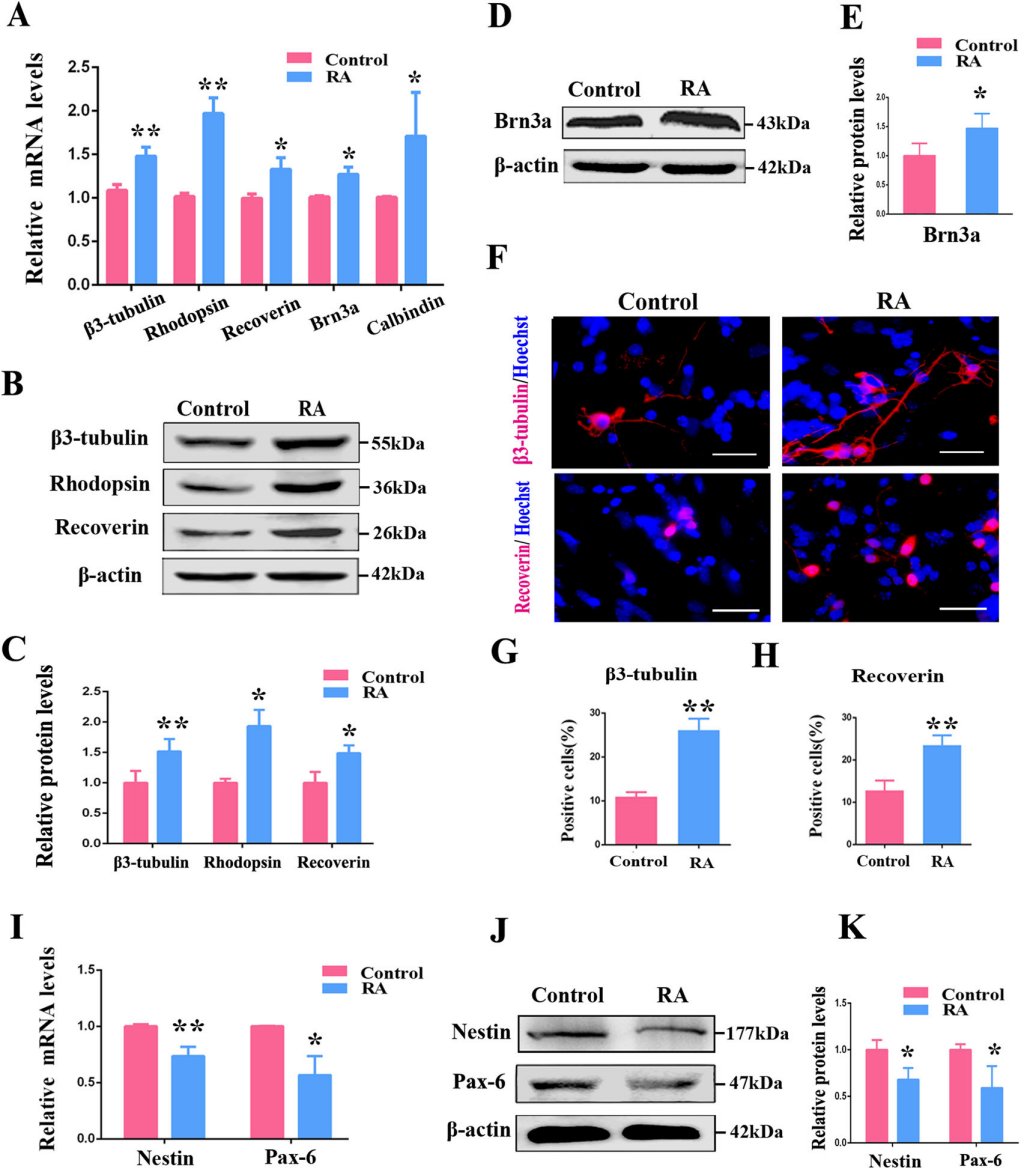
RA enhances RPC differentiation. **a** The qPCR analysis showed that the expression levels of the RPC differentiation-related markers, including β3-tubulin, rhodopsin, recoverin, Brn3a and calbindin, were elevated in RA-treated RPC cultures. **b**-**e** The results of the western blot revealed that β3-tubulin, rhodopsin, recoverin and Brn3a were upregulated in RA-treated RPC cultures compared with the control. The western blot was scanned and normalized to β-actin. **f**-**h** Immunochemistry showed that the percentages of β3-tubulin- and recoverin-positive cells in RA group were significantly higher than those in control group. **i**-**k** According to the qPCR and western blot, the expression levels of nestin and Pax-6 were repressed in the RA-treated RPC cultures. Scale bars: 50 μm. Data are the averages of three independent experiments. Error bars indicate the standard deviation of the mean. *P ≤ 0.05, **P ≤ 0.01 (Student’s* t*-test)

To determine the effect of RA on the differentiation of RPCs, the RPCs were treated with RA for 7 days under differentiation conditions. The expression levels of differentiation markers β3-tubulin, rhodopsin, recoverin, Brn3a and calbindin were enhanced in RA-treated RPCs, according to the results of the qPCR and western blot (Figs. 4a-e). Additionally, the proportions of β3-tubulin- and recoverin-positive cells were increased in RA-treated RPC cultures when compared with the control group (Figs. 4f-h). Although nestin and Pax-6 showed an opposite trend, decreasing compared with the control group (Figs. 4i-k). Collectively, these data showed the effects of RA on RPC growth, which generally had the same tendencies as the knockdown of REST expression; that is, it inhibited proliferation but promoted differentiation, suggesting a negative correlation of REST and RA.

### RA downregulates the expression level of REST

**Fig. 5:**
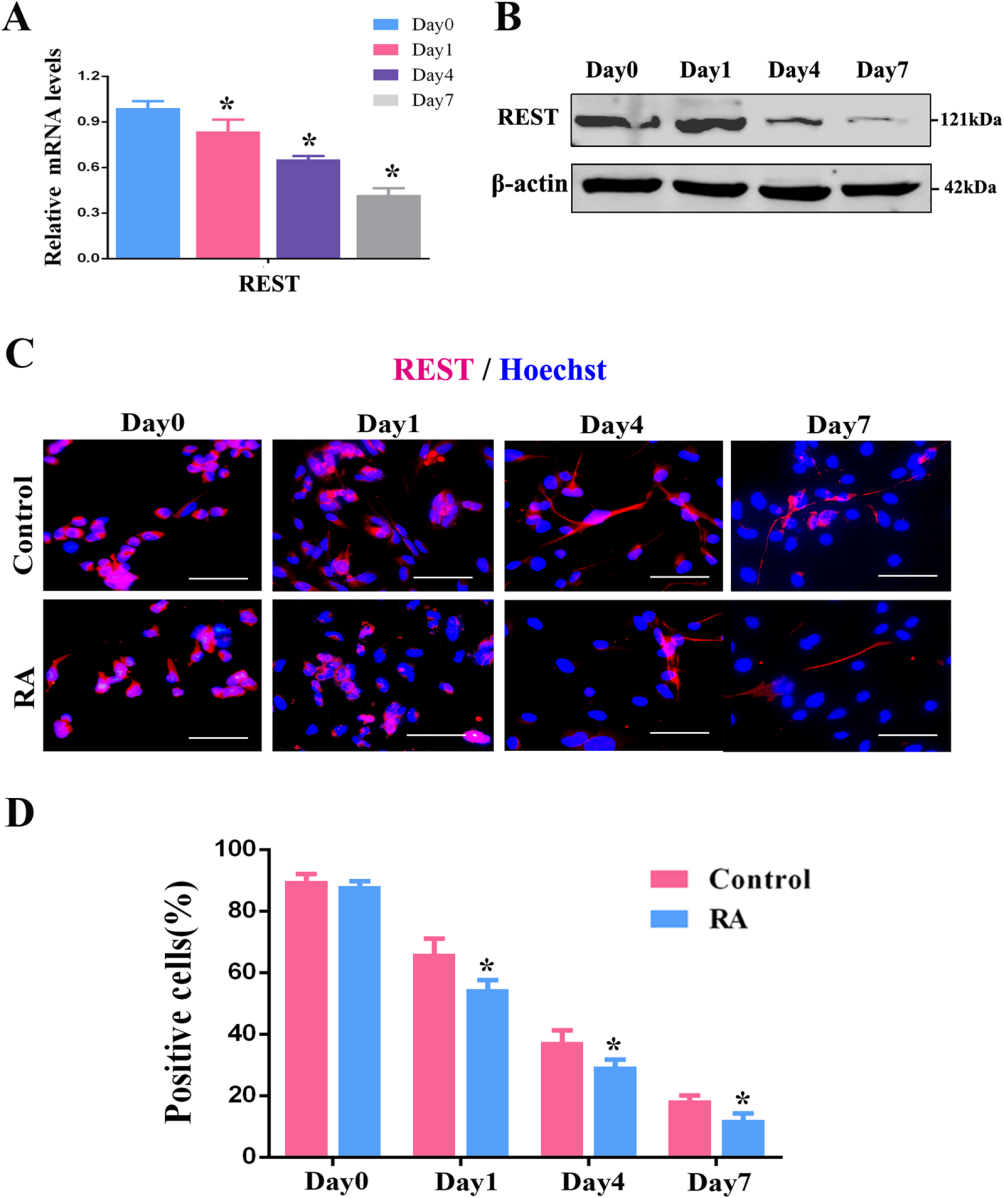
RA downregulates REST protein expression. **a** The qPCR and **b** western blot results revealed that REST was gradually decreased during RPC differentiation. **c**, **d** Immunocytochemistry showed that RA accelerated the decrease of REST protein levels during RPC differentiation. Data are the averages of three independent experiments. Scale bars: 50 μm. Error bars indicate the standard deviation of the mean. *P ≤ 0.05 (Student’s* t-*test and one-way ANOVA)

According to the above results, RA inhibited the proliferation but promoted the differentiation of RPCs, in agreement with the siREST results. In the current study, we first detected the endogenous expression of REST during RPC differentiation. As the qPCR analysis disclosed, in comparison with day 0, the expression levels of REST gradually decreased during RPC differentiation, dropping to approximately 40% at day 7 (Fig. 5a). Notably, the REST protein level also gradually decreased over time in the differentiation medium (Fig. 5b). To verify our speculation that RA is involved in the modulation of REST expression, RPCs were induced to differentiate with RA for 7 days. As the immunocytochemistry analysis showed, REST gradually decreased during differentiation, and the RA treatment could accelerate this reduction process (Figs. 5c, d). These results suggested that RA enhanced the downregulation of REST expression during RPC differentiation.

### REST regulated by RA through miR-29a and proteasomal degradation

**Fig. 6:**
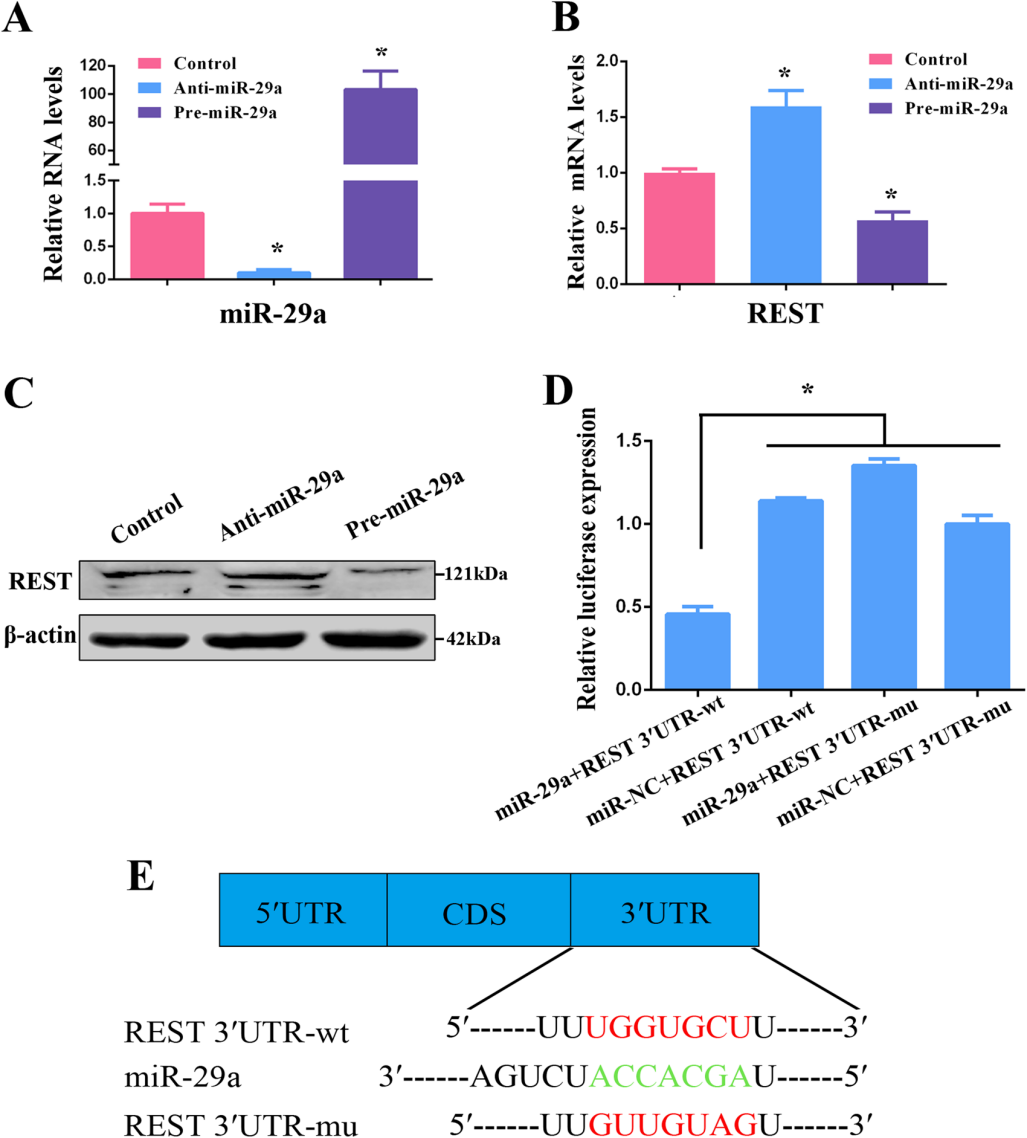
REST is a target gene of miR-29a in RPCs. **a** The qPCR analysis showed that compared with control group, the expression levels of miR-29a were sharply downregulated by miR-29a inhibitor, and remarkably upregulated by miR-29a mimics. **b, c** According to the qPCR and western blot results, the REST expression level was downregulated by the overexpression of miR-29a but promoted by transfection with the miR-29a inhibitor. **d, e** Positions 561–567 of the 3′-UTR of the REST mRNA (REST 3′-UTR-wt) or a mutated (REST 3′-UTR-mu) sequence was designed and inserted into the pGL3-control plasmids. The dual luciferase reporter system confirmed miR-29a binding to the wild-type 3′-UTR sequences of REST. Co-transfection of miR-29a and the REST wild-type 3′-UTR binding site (REST 3′-UTR-wt) dramatically reduced the luciferase activity compared with the other groups. The firefly luciferase activity data were normalized to Renilla luciferase activity as a control. Data are the averages of three independent experiments. Error bars indicate the standard deviation of the mean. *P ≤ 0.05 (Student’s* t-*test and one-way ANOVA)

Based on the above results, we further explored how RA downregulated REST expression. A previous study disclosed that RA regulated neural gene expression through altering miRNA expression^44^. Meanwhile, REST was the target gene of miR-29a^35^. Thus, we speculated that, in RPCs, REST also interacted with miR-29a and that the regulation of REST by RA might involve miR-29a expression. The following experiment was first performed to detect whether miR-29a regulates the expression of REST in RPCs. In the current study, RPCs were transduced with the miR-29a mimics (pre-miR-29a group) or inhibitor (anti-miR-29a group) in proliferation medium. The transfected efficiency of miR-29a was detected by qPCR (Fig. 6a). As shown in Fig. 6b, in the miR-29a mimic- and miR-29a inhibitor-transfected RPCs, REST was significantly transcriptionally suppressed and transcriptionally stimulated, respectively, suggesting that miR-29a negatively regulated the expression of REST. The results of western blot also displayed that compared with the control group, the expression level of REST was downregulated by the miR-29a mimics but upregulated by the miR-29a inhibitor, respectively (Fig. 6c). These results indicate REST may be an active target gene of miR-29a in RPC growth. To further verify that miR-29a binds to the REST mRNA 3′-UTR to regulate the translational progress, a luciferase reporter was performed. REST 3′-UTR containing the miR-29a binding site (REST 3′-UTR-wt) and its mutant (REST 3′-UTR-mu) at the positions 561–567 were cloned downstream of the firefly luciferase coding sequence in the pGL3-control vector. The luciferase assay showed that the luciferase activity was dramatically decreased during co-transfection of miR-29a and REST 3′-UTR-wt compared with the other groups (Fig. 6d), indicating that positions 571–576 of the REST 3′-UTR were a direct target of miR-29a (Fig. 6e). These data indicate that miR-29a regulates REST expression by directly binding to the REST 3′-UTR.

**Fig. 7:**
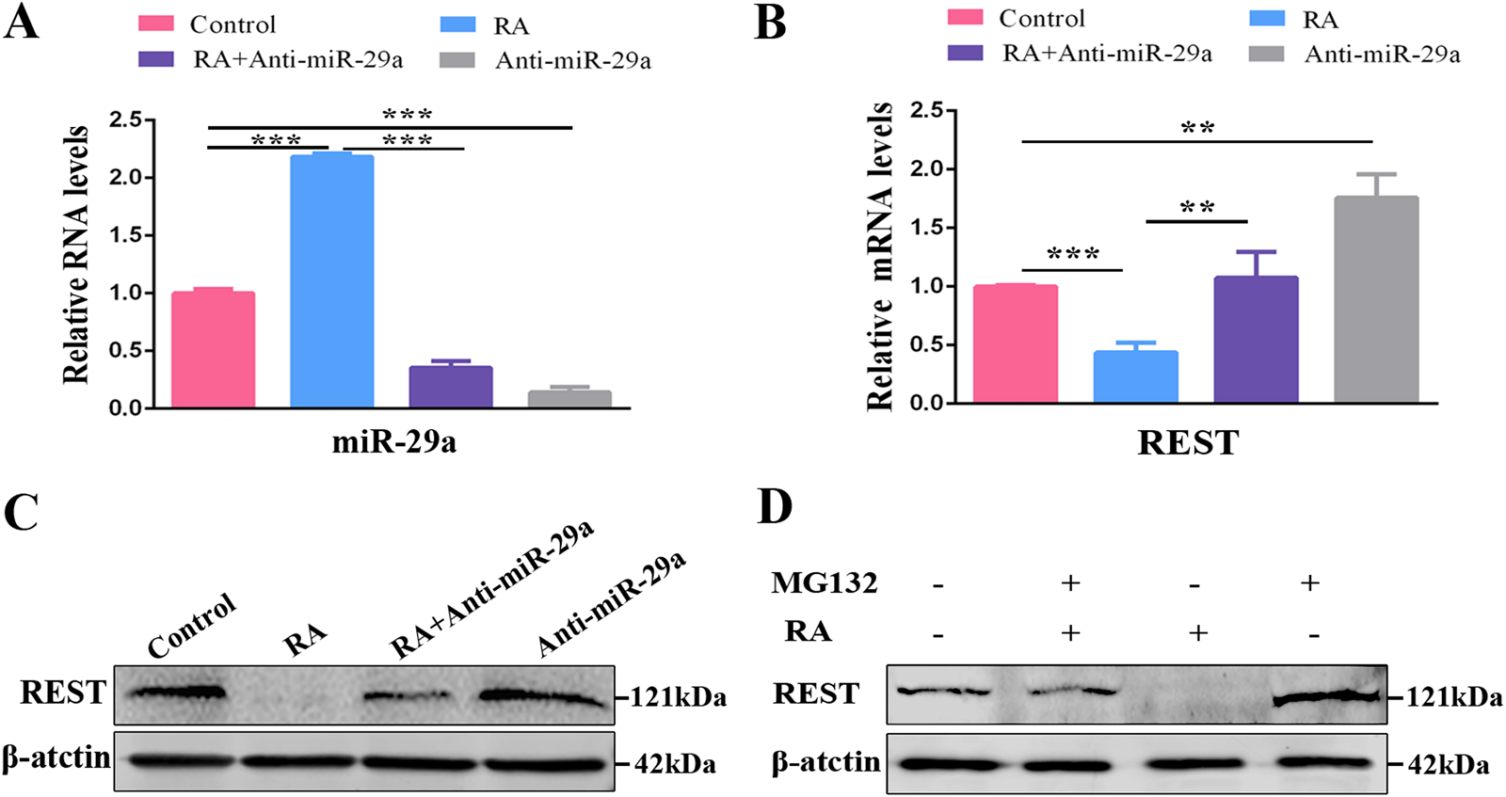
RA inhibits REST expression through miR-29a and proteasome. **a** The expression level of miR-29a was remarkably elevated in RA-treated RPC cultures (compared with the control group), and this upregulation could be crippled by miR-29a inhibitor. **b,c** The qPCR analysis and western blot revealed that REST expression level was decreased in RA group (compared with the control group), and this downregulation could be rescued by miR-29a inhibitor. **d** The western blot showed the expression level of REST was reduced significantly in RA-treated RPC cultures (lane 3) compared with control (lane 1); this reduction could be reversed by MG-132 treatment (lane 2). Data are the averages of three independent experiments. Error bars indicate the standard deviation of the mean. **P ≤ 0.01, ***P ≤ 0.001 (Student’s *t*-test)

We next tested whether RA can regulate the expression of miR-29a and further alter the REST expression level. RPCs were cultured under proliferation medium without (control group) or with RA (RA group), RA plus miR-29a inhibitor (RA + miR-29a inhibitor group) or miR-29a inhibitor (miR-29a inhibitor group) for 3 days. It was worth noting that, as we expected, according to the qPCR analysis, miR-29a was upregulated remarkably in the RPC cultures treated only with RA compared with the control group, and this can be reversed by adding the miR-29a inhibitor (Fig. 7a), indicating that RA promotes the expression of miR-29a and that this promotion could be blocked by the miR-29a inhibitor. Conversely, the expression of REST was downregulated to 50% in RA-treated RPCs compared with the control, and this suppression could be reversed by the miR-29a inhibitor (Fig. 7b). The western blot analysis was consistent with the qPCR results (Fig. 7c). The above data suggest that REST could be mediated by RA through upregulation of miR-29a.

Importantly, the expression of REST in the culture treated with both RA and miR-29a inhibitor (Fig. 7c—lane 3) was significantly more than that in the RPC cultures treated only with RA (Fig. 7c—lane 2) but still less than that in the control (Fig. 7c—lane 1), indicating that other mechanisms might be involved in this process. In general, there are two main pathways to downregulate REST protein expression, decrease its generation (transcriptional level) and accelerate its degradation (posttranscriptional level). We then treated RPCs with the proteasomal inhibitor MG-132 (20 µM) for 8 h in the presence and absence of RA (under proliferation conditions). The protein level of REST was significantly rescued by MG-132 (in RPC cultures treated with both RA and MG-132, Fig. 7d—lane 2) compared the cells treated with RA alone (Fig. 7d—lane 3), demonstrating that blocking the proteasomal activity could counter the RA-mediated degradation of the REST protein.

**Fig. 8:**
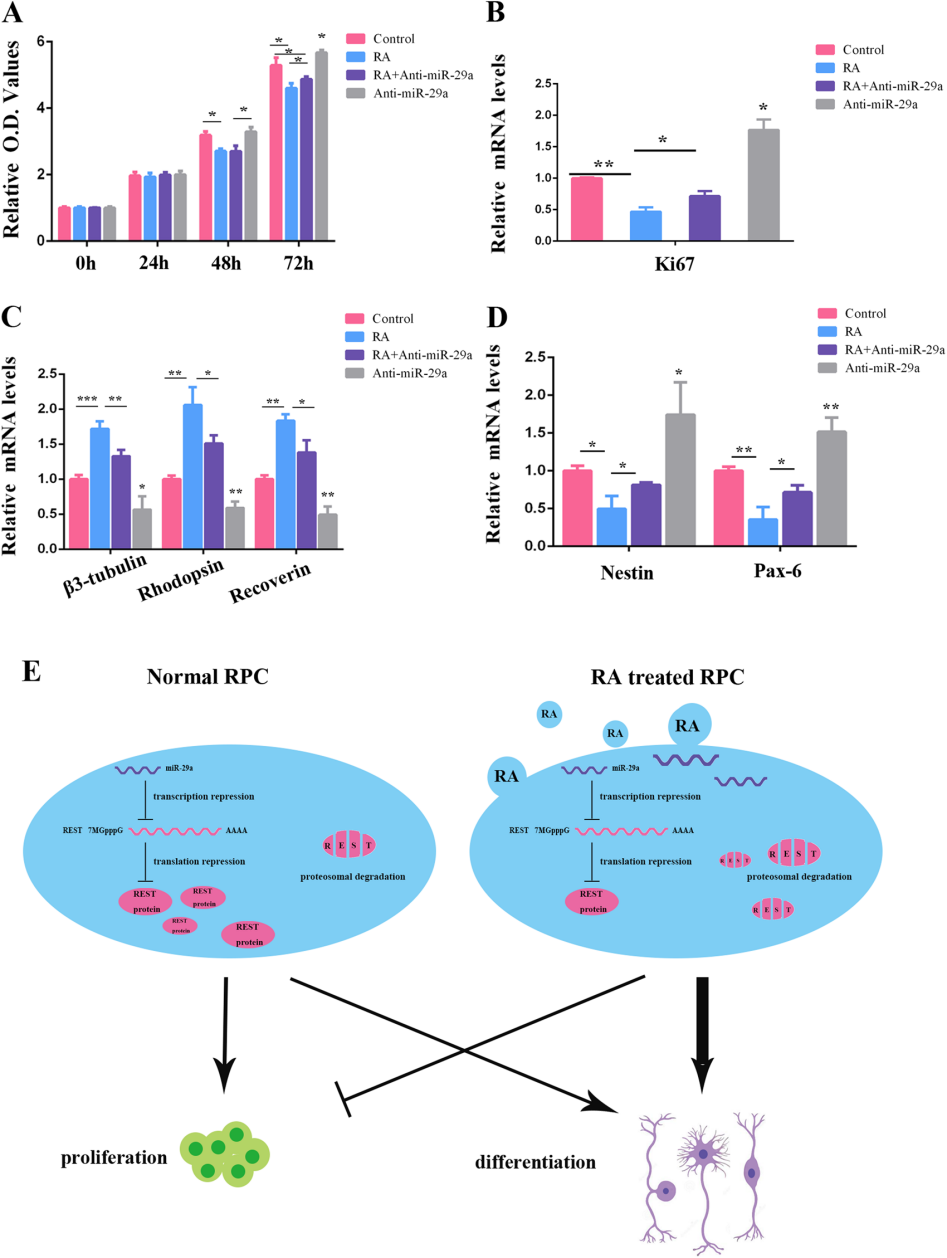
RA regulates RPC proliferation and differentiation through miR-29a. **a** The results of the CCK-8 analysis showed that RA induced RPC proliferation inhibition, and this trend could be partially rescued by the miR-29a inhibitor. **b** The qPCR analysis showed that the expression of Ki-67 was upregulated in RPCs treated with both RA and the miR-29a inhibitor compared with those only treated with RA. **c** The expression levels of RPC differentiation markers (β3-tubulin, rhodopsin and recoverin) were increased RA-treated RPC cultures (compared with control cultures), and miR-29a inhibitor partially reversed RA induced RPC differentiation markers upregulation. **d** Compared with RPCs only treated with RA, the expression levels of nestin and Pax-6 were upregulated in RPCs treated with both RA and the miR-29a inhibitor, according to the qPCR analysis. **e** A model of the role of REST mediated by RA in the regulation of RPC proliferation and differentiation. RA could directly degrade REST protein through the proteasome and indirectly suppress REST gene expression through the upregulation of miR-29a. Data are the averages of three independent experiments. Error bars indicate the standard deviation of the mean. *P ≤ 0.05, **P ≤ 0.01, ***P ≤ 0.001 (Student’s *t*-test)

To further determine whether the effect of RA on RPC proliferation and differentiation was through miR-29a, CCK-8 and qPCR analyses were performed. In proliferation medium, the RA-induced RPC proliferation inhibition could be partially rescued by the miR-29a inhibitor, according to the CCK-8 analysis (Fig. 8a). The qPCR analysis showed that the expression level of the cell proliferation marker Ki-67 was suppressed by approximately 50% in the RA group compared with the control group, and this suppression was rescued partially by the miR-29a inhibitor (in RA + miR-29a inhibitor group, Fig. 8b). Additionally, RA-induced neuronal differentiation promotion (under differentiation medium) was also crippled by adding miR-29a inhibitor (Fig. 8c). Furthermore, compared with RPCs only treated with RA, the expression levels of nestin and Pax-6 were also upregulated in RPCs treated with both RA and the miR-29a inhibitor, according to the qPCR analysis (Fig. 8d). Therefore, we concluded that RA inhibited the proliferation and induced the differentiation of RPCs partly through promoting miR-29a expression.

Taken together, our data suggest that REST can enhance proliferation of RPCs but restricts their differentiation abilities and that the expression of REST can be mediated by RA at both the transcriptional and posttranscriptional levels (Fig. 8e).

## Discussion

RPCs are a subset of multipotent cells that are characterized by capacities of self-renewal and differentiation into retinal neurons. As a promising resource for cell-based therapy in the treatment of retinal degenerative diseases, such as AMD and RP, the primary obstacles of RPCs currently are their limited proliferation and differentiation ability into specific retinal neurons in vitro^45,46^. In this study, our results indicate that REST is a conserved, neuron-suppressed protein that could be modulated by RA through miR-29a and proteasomal degradation, respectively, to regulate the proliferation and differentiation of RPCs.

REST has been recognized as a repressor of neuronal genes during neurogenesis^47^. It is known to be expressed in the nuclei of different types of cells and bind RE-1 to restrain terminal neuronal differentiation-related genes^47–49^. Thus, REST was present at its highest level in the nuclei of pluripotent ESCs and was gradually downregulated and translocated into the cytoplasm with the maturation of neurons^13,50^. In this study, REST was also found to be highly expressed in nuclei in proliferation medium and translocated into the cytoplasm when it was downregulated during RPC differentiation. In ESCs and NPCs, REST played a role in maintaining their multipotency and the direct knockdown of REST to promote differentiation^11,42^. Our study demonstrated the self-renewal abilities of RPCs, which were also impaired after knocking down REST expression, whereas the marker expression for retinal neurons was upregulated. In addition, the percentages of nestin-positive cells in siREST-treated RPC cultures exhibited no significant change, although the immunoreactive intensity of nestin faded (Fig. S1), implying that interference of REST expression did not result in a loss of RPC multipotency, which is consistent with a previous study^42^. These results indicated that REST in RPCs, as in ESCs and NPCs, plays an important role in maintaining cell proliferation and restricting their differentiation.

As direct knockdown of endogenous REST reduced the RPC proliferation but accelerated the RPC differentiation, which phenocopied the observed effects of RA on RPCs, we speculated that RA is involved in REST regulation. Our results showed that the REST protein levels could be significantly downregulated by RA, and two mechanisms were involved in this process, including decrease in the production and promotion of the degradation of REST protein. In ESCs, previous study reported that RA regulated mRNA transcription by suppressing the expression levels of miR-200b and miR-200c^44^. In this study, we showed, in RPCs, that RA could inhibit REST mRNA expression partly through enhancing the expression of miR-29a (the luciferase report verified that REST is a target gene of miR-29a, consistent with a previous study^35^). Our further study showed that RA-induced RPC proliferation inhibition and differentiation promotion could be crippled by the miR-29a inhibitor, which, in turn, negatively mediated REST expression, implying that RA worked on RPCs through miR-29a. It is known that a miRNA usually has multiple target genes, Zhang et al. reported that miR-29a could promote RPC differentiation and attenuated RPC proliferation by negatively regulating the expression of RBM8a (one target gene of miR-29a)^37^, indicating that RBM8a may have a role in RA regulating RPC proliferation and differentiation. In addition, REST was also reported to be regulated by RA through a posttranscriptional mechanism mediated by proteasomal degradation^51^, and RA can upregulate the expression level of REST-specific E3-ligase (Skp1-Cul1-F-box protein complex containing the F-box protein β-TRCP, SCFβ-TRCP), which promote REST proteasomal degradation in neuroblastoma cells^12,51^. In the current study, REST protein levels were substantially increased in the presence of the proteasome inhibitor MG-132 plus RA group compared with the levels in the cells treated with RA alone. The reversal of RA-mediated REST degradation by MG-132 treatment indicates the proteasomal involvement in modulating the REST levels in RPCs. However, other mechanisms may be also involved in RA regulating RPC proliferation and differentiation. Previous studies displayed that RA promoted neural differentiation at the expense of proliferation through upregulated the proneural and neurogenic transcription factors^52,53^, but downregulated pro-proliferation transcription factors^54–57^. RA may also work on its receptors (RARs) to regulate the direct downstream target genes, which are involved in the cell proliferation–differentiation switch^53,58,59^. Taken together, our data demonstrated that RA induced REST suppression through miR-29a-modulated transcriptional and proteasome-modulated posttranscriptional levels. RA, as a recognized secure and effective clinical medicine in the treatment of various diseases, is supposed to be applied in future retinal degeneration therapy.

This study discloses a novel functional protein, REST, which governs RPC proliferation and differentiation, can be mediated by RA, which may be instructive for the application of RPC transplantation in the treatment of retinal degeneration diseases. Further studies will focus on elucidating the roles of REST and RA in retinal development and their application in vivo to treat retinal degeneration diseases.

## Conclusion

In this study, our data demonstrated that REST plays an important role in RPC proliferation and differentiation. Knockdown of REST expression inhibits proliferation of RPCs but stimulates their differentiation into specific retinal neurons, implying that REST can be proposed as a novel biomarker for evaluating RPC behavior. Another key finding is that REST expression could be regulated at the transcriptional and posttranscriptional levels by RA through elevating miR-29a expression and accelerating proteasomal degradation, respectively. The current study provides us with new sights in controlling RPC fates, although the effects of REST in vivo remain to be explored.

## Electronic supplementary material

figureS1(TIF 712 kb)

figureS2(TIF 774 kb)

Supplemental material(DOCX 16 kb)
