# Supplementary figures and images for: REST, regulated by RA through miR-29a and the proteasome pathway, plays a crucial role in RPC proliferation and differentiation
Source: Cell Death Dis. 2018 Apr 18;9(5):444. doi: 10.1038/s41419-018-0473-5 (PMC5906654; doi:10.1038/s41419-018-0473-5)

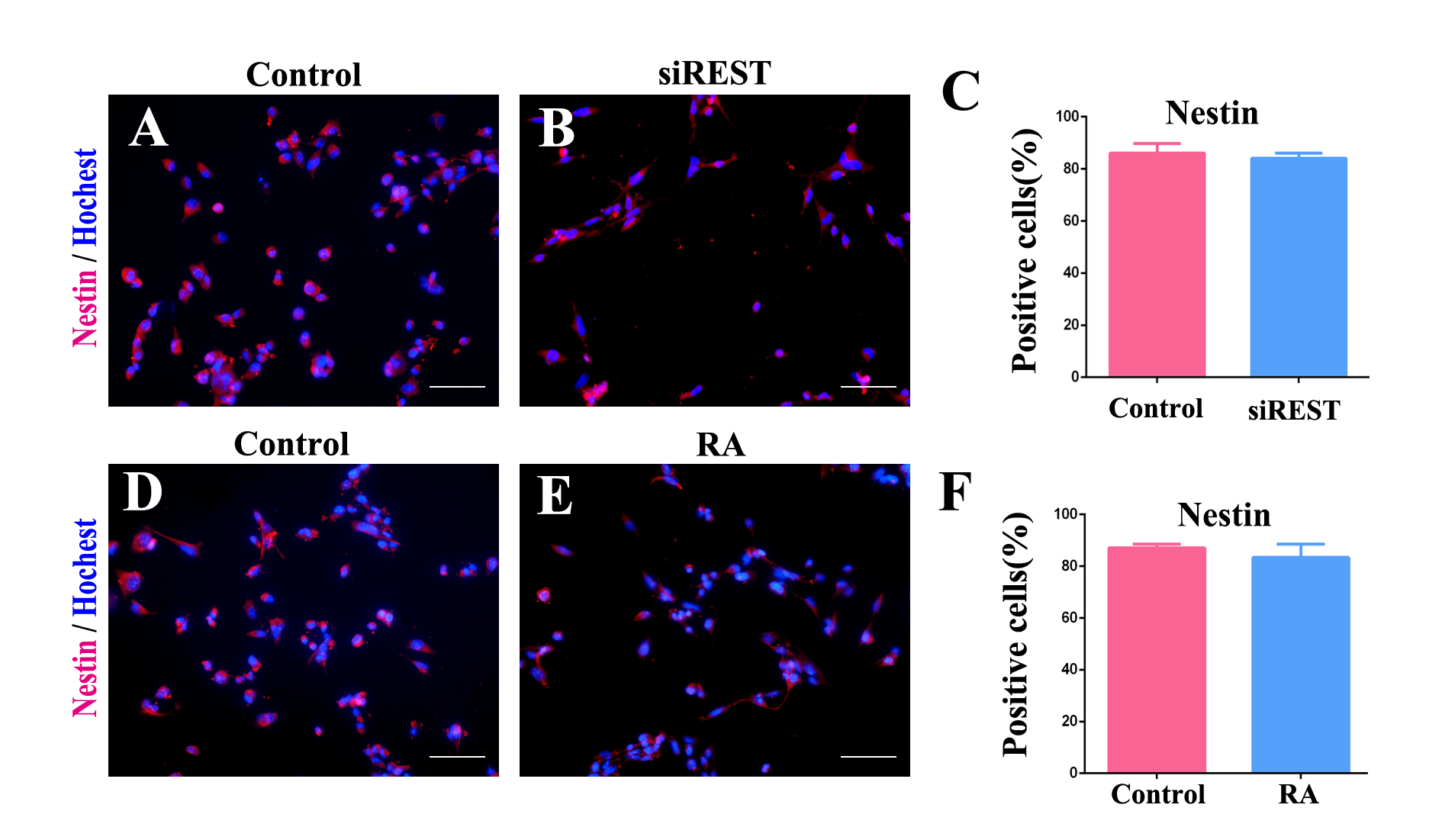

Supplement: Supplementary file 1 — figureS1(TIF 712 kb) [file 41419_2018_473_MOESM1_ESM.tif]

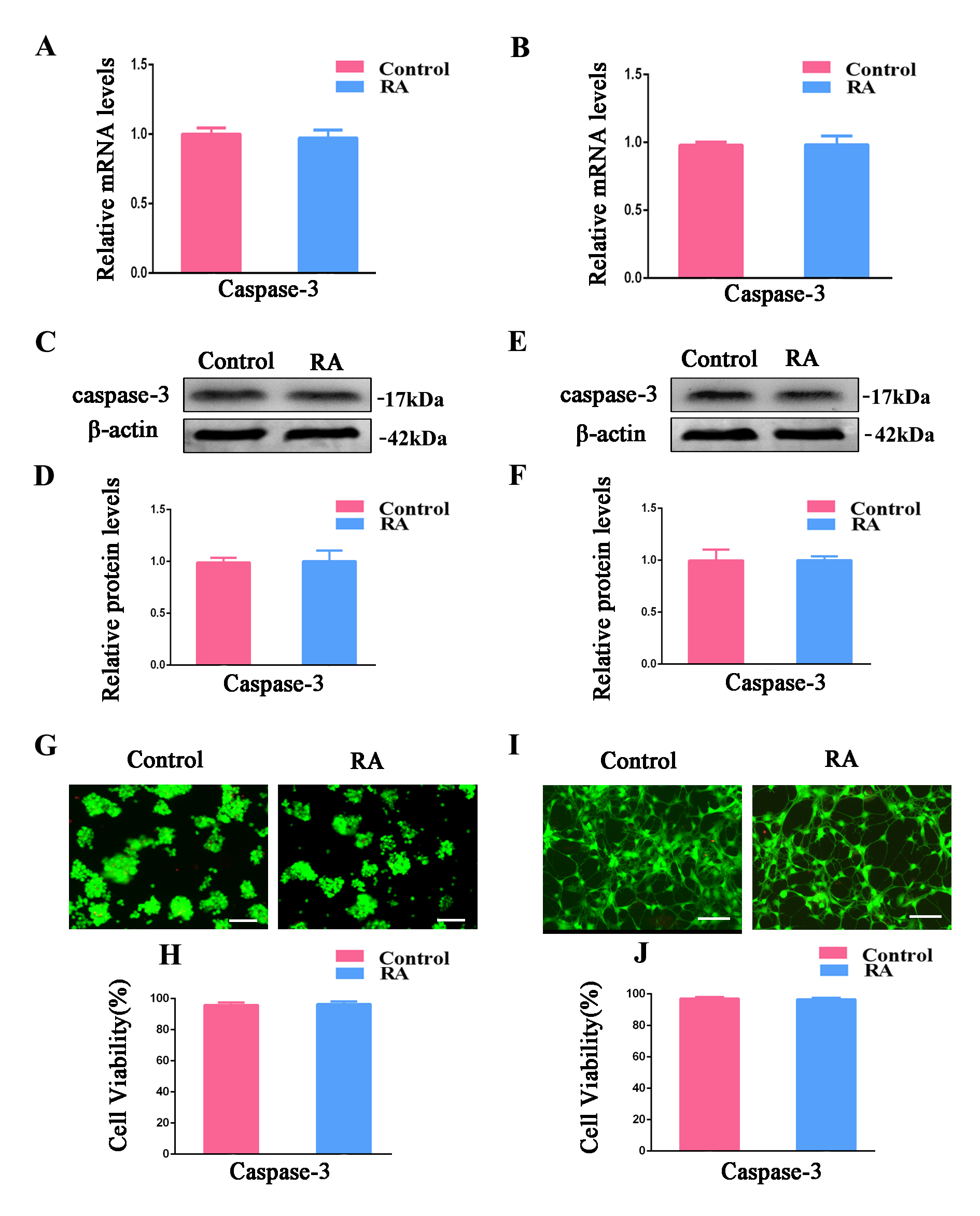

Supplement: Supplementary file 2 — figureS2(TIF 774 kb) [file 41419_2018_473_MOESM2_ESM.tif]
