# Supplementary material for: REST, regulated by RA through miR-29a and the proteasome pathway, plays a crucial role in RPC proliferation and differentiation
Source: Cell Death Dis. 2018 Apr 18;9(5):444. doi: 10.1038/s41419-018-0473-5 (PMC5906654; doi:10.1038/s41419-018-0473-5)
Supplement: Supplementary file 3 — Supplemental material(DOCX 16 kb) [file 41419_2018_473_MOESM3_ESM.docx]

**Supplementary Figure Legends**

**Figure S1.** siREST and RA do not change RPC multipotency. Under proliferation conditions, the retinal progenitor marker nestin was evaluated by immunostaining analysis. No significant difference of the percentage of nestin-positive cell was observed in siREST (A-C) or RA (D-F) treated RPC cultures compared with control cultures. Scale bars: 100 μm. Data are the averages of three independent experiments. Error bars indicate the standard deviation of the mean (Student’s t-test).

**Figure S2.** RA does not induce RPC apoptosis. (A-B) The qPCR analysis showed that there is no significant difference of the expression levels of cell apoptosis marker caspase-3 between RA group and control group during RPC proliferation (A) and differentiation (B). (C-F) The Western bolt results showed no obvious difference of caspase-3 protein levels in RA group (compared with the control group) in proliferation (C-D) and differentiation (E-F) medium. (H-J) Live/Dead assay displayed that only a few dead cells were detected in both RA group and control group under proliferation (G-H) and differentiation (I-J) medium. Scale bars: 100 μm. Data are the averages of three independent experiments. Error bars indicate the standard deviation of the mean (Student’s t-test).
